# Ceramic Onlay: A Case Report

**DOI:** 10.7759/cureus.32641

**Published:** 2022-12-17

**Authors:** Sravani Nirmala, Srikanth Goud G, Naresh Kumar K, Rakesh Reddy Chukka, Narender Reddy

**Affiliations:** 1 Conservative Dentistry and Endodontics, SVS Institute of Dental Sciences, Mahabubnagar, IND; 2 Oral Medicine and Radiology, SVS Institute of Dental Sciences, Mahabubnagar, IND

**Keywords:** resin cement, composites, aesthetics, strength, tooth, direct restoration, indirect restoration, adhesive cements, onlays, ceramics

## Abstract

With the growing demand for minimally invasive dental restoration, conservative treatment options offer good aesthetic outcomes and durability with minimal tooth reduction. The use of ceramics in dental restoration has been on the rise in the past two decades due to its long-term benefits and capacity to produce a superior bond to tooth structure when used in conjunction with adhesive cement and acid-etch procedures. A ceramic onlay, which covers one or more cusps, also helps to preserve dental tissue. These materials enable the dentist to conservatively prepare the tooth for restoration while also strengthening the patient’s own tooth structure. This case report discusses the preparation of a functional and aesthetic ceramic onlay for a patient with a cracked tooth.

## Introduction

Dental restorations can be direct (i.e., the procedure is conducted entirely in the patient’s oral cavity without the use of a dental laboratory) or indirect (i.e., the material is prepared in a lab and then inserted into the patient). Current research does not definitively indicate as to which approach is preferable since many factors affect this decision, such as the structure and location of the missing tooth, arch structure, status of the dental pulp, nature of the occlusion, patient concerns, and dentist preference. Among all these factors, the amount of remaining tooth structure and its strength play a significant role. As advancements in adhesive materials lessen the need for substantial tooth tissue removal and as rates of dental caries continue to decline, it is important to develop more conservative cavity treatments [1].

Partial restorations are rapidly replacing conventional crowns as the demand for minimally invasive restorations increases [2]. These restorations include ceramic, composite, and metallic (e.g., gold, nickel, chrome) inlays and onlays, as well as resin-bonded minimal preparation bridges and bonded ceramic bridges. Onlays are bonded restorations with supragingival borders and cuspal coverage to preserve tooth structure. Onlays do not cover the entire external structure and thus can simplify the tooth preparation, impression-making, cementation, finishing, and polishing processes. Biocompatible ceramic onlays are the material of choice in modern dentistry. Compared to resinous materials, ceramic offers physical and mechanical characteristics more similar to enamel and dentine, such as a higher modulus of elasticity, hardness, and coefficient of thermal expansion [3]. Modern adhesives also provide superior bonding, even though proper tooth preparation should not be ignored [4].

Complications of partial restorations include fractures, tooth sensitivity, poor adjustment, marginal integrity loss, microleakage, and adhesion failure. Other factors that affect clinical performance include plaque accumulation, gingivitis, secondary caries, color instability, anatomical shape, radiopacity, material wear, and wear on adjacent teeth. Other variables such as the state of supporting teeth, patient habits, clinical procedures, and characteristics of the restorative material also affect the survival and durability of the onlay [2]. This case study describes an indirect partial restoration of a tooth in a 51-year-old male patient using a ceramic onlay.

## Case presentation

**Figure 1:**
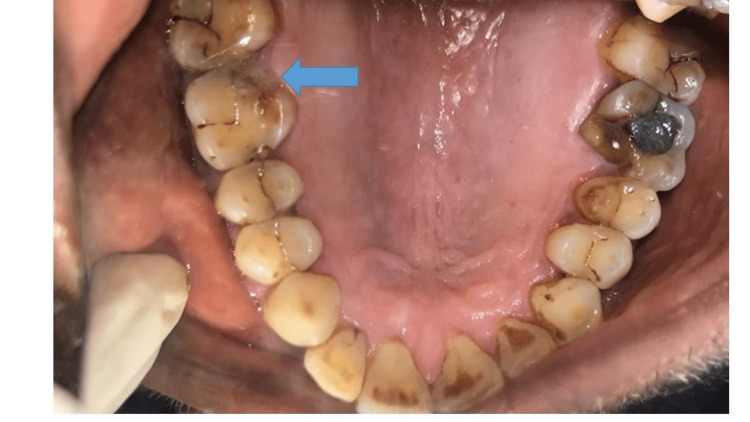
Preoperative intraoral view

**Figure 2:**
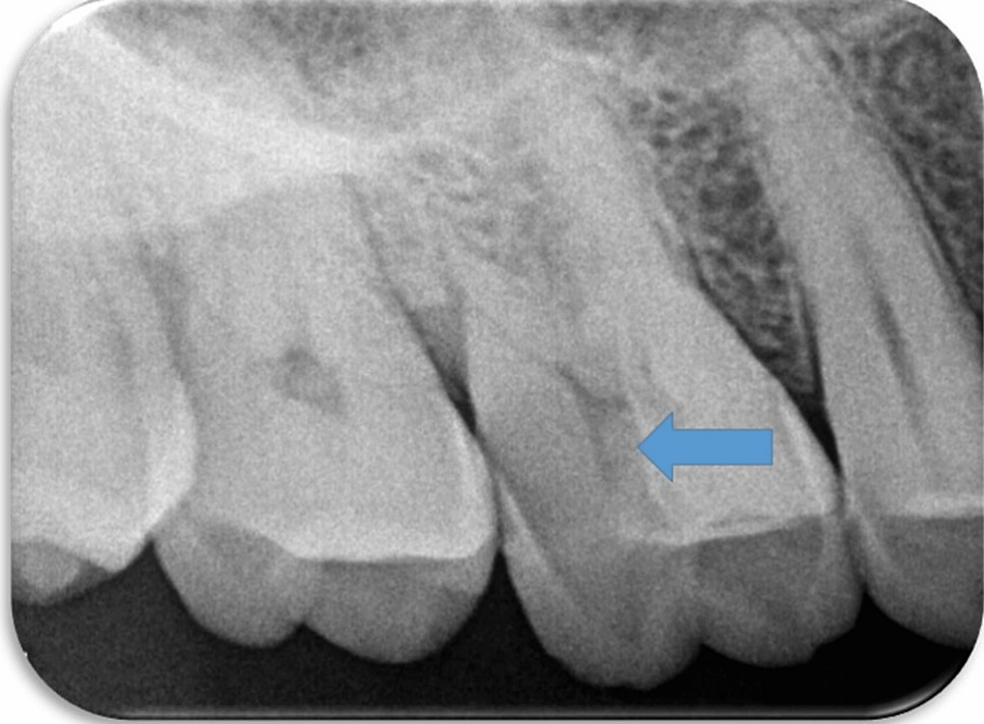
Preoperative radiographic image

A 51-year-old man presented to the department of conservative dentistry and endodontics with a chief complaint of cracked tooth and food lodgment in the area of the upper right back teeth for two months. The clinical examination and radiographic evaluation such as radiovisiography using a sensor were taken in mesial, distal, and straight angulation, which, along with occlusal radiographs, revealed distoproximal caries with mesiopalatal and distopalatal cuspal fracture in connection to tooth 16 (Figures 1, 2) and root canal-treated tooth with post endo amalgam restoration in connection to 26. On radiographic evaluation, there was radiolucency involving enamel and dentin indicating distoproximal caries with mesiopalatal and distopalatal cuspal fracture in relation to 16 (Figure 2). After examination, a treatment plan was discussed in relation to 16.

**Figure 3:**
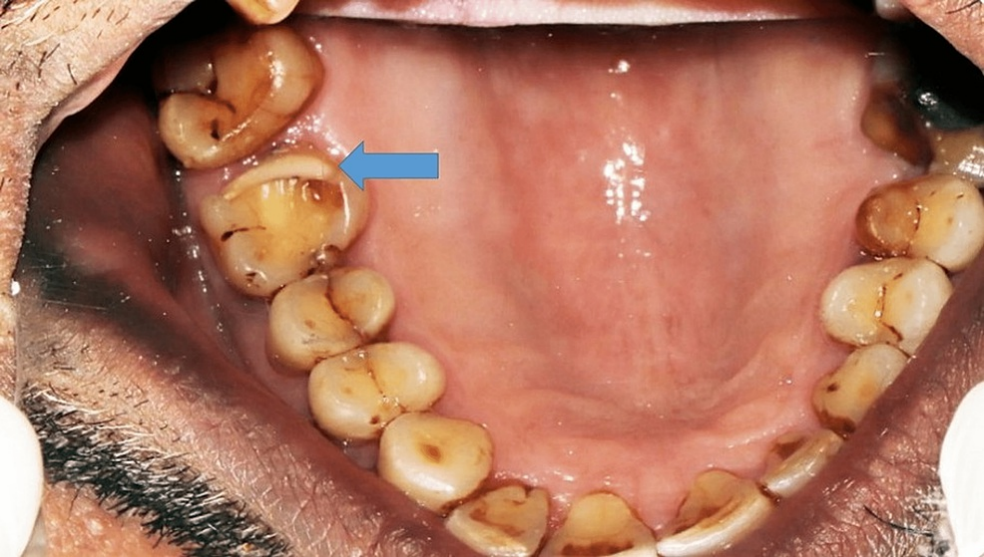
Cavity preparation

To address the patient’s concerns about the aesthetics of the tooth, we planned a ceramic onlay restoration for tooth 16. The cavity was prepared according to the principles of onlay cavity preparation (Figure 3) [5]. The total decrease was 2 mm, including the occlusal work on the mesiopalatal and distopalatal cusps and the distoproximal gingival finish line using 271 and 169L carbide burs. All line and point angles were rounded using 8862 flame-shaped diamond bur to reduce the concentration of internal tensions. The adjacent tooth had a 0.5-mm proximal clearance. A divergence of approximately 10° between opposing walls was established and done using 271 carbide bur. This divergence gives a passive insertion axis for ceramic restorations without unnecessarily destroying the healthy tooth structure. The cervical margin was finished with a deep chamfer, and the cavosurface angles were made at 90°, as previously described [5].

**Figure 4:**
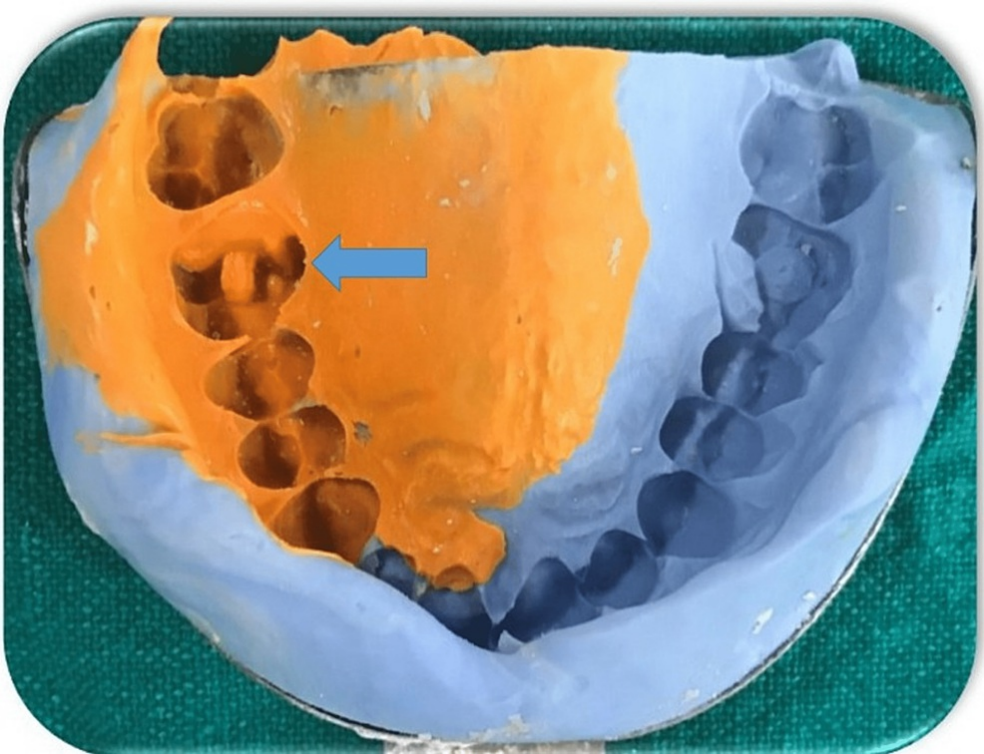
Putty impression of the preparation

After cavity preparation, the cavity margins were examined thoroughly. A putty impression was made using heavy body and light body impression material using a single technique (Aquasil Soft Putty, Dentsply Sirona, Charlotte, NC) to obtain fine details of the prepared cavity. The impression was sent to the laboratory for ceramic onlay fabrication (Figure 4).

**Figure 5:**
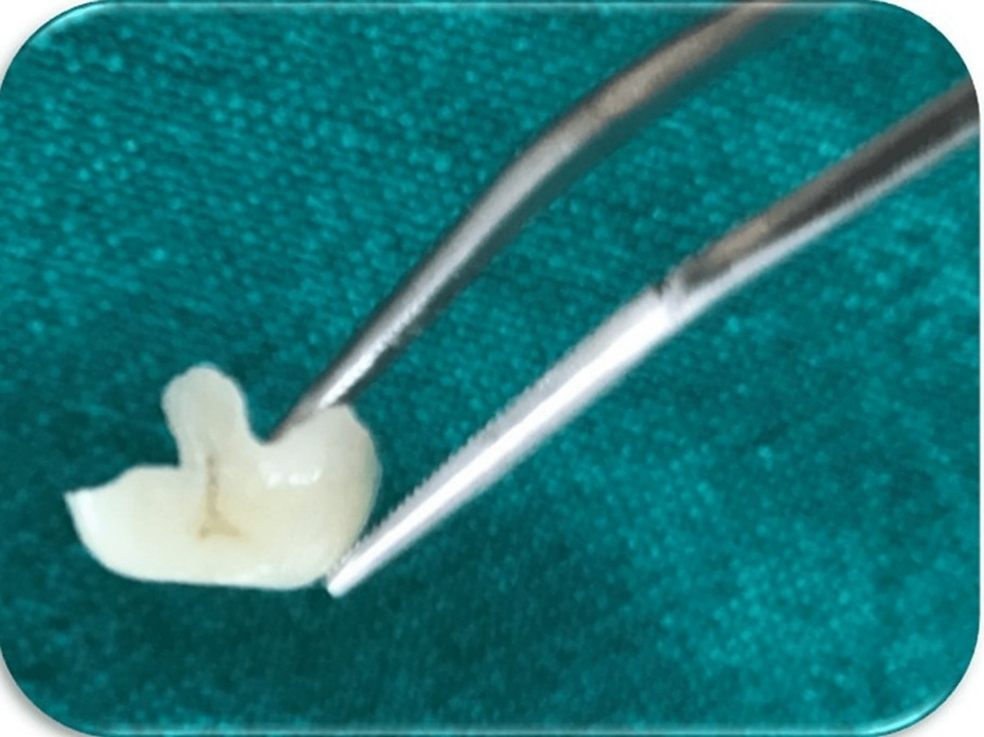
Ceramic onlay preparation from the laboratory

In the laboratory, the cast was prepared, scanned, and milled using a CEREC Zirconia block (Dentsply Sirona). The prepared onlay was examined for polish texture and inconsistencies before inserting into the patient's cavity (Figure 5).

**Figure 6:**
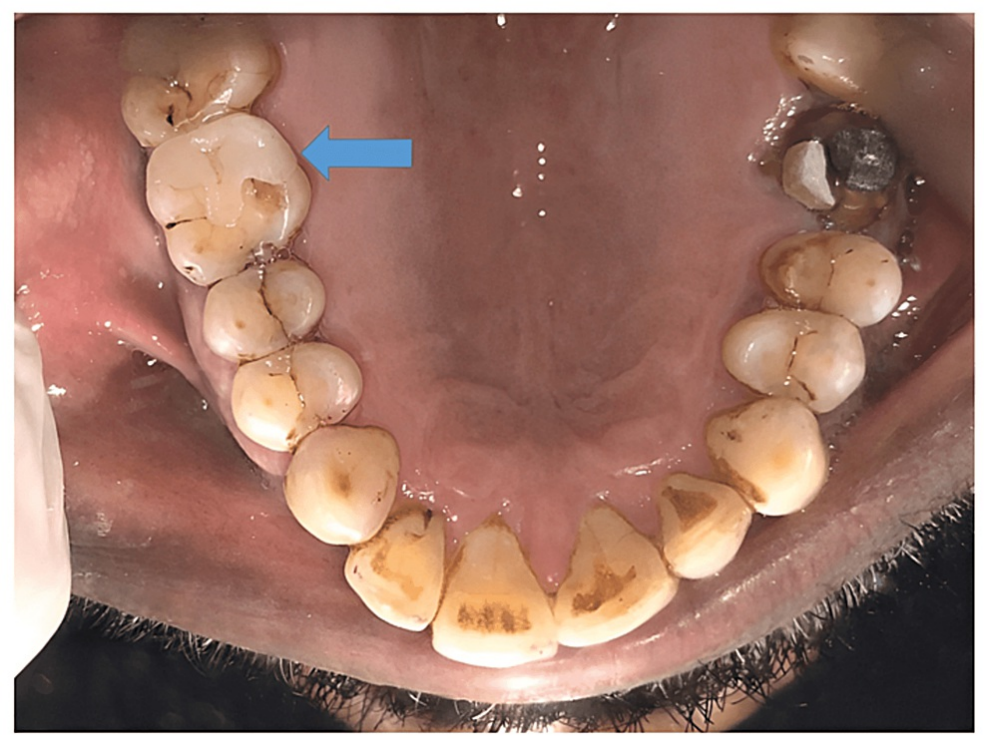
Ceramic onlay cementation

To prepare the ceramic surface for bonding, 9.5% buffered hydrofluoric acid (Porcelain Etch, Ultradent Products, Provo, UT) was applied for one minute. The surface was then rinsed with water and allowed to air dry. A mini sponge was used to apply a silane coupling agent (Rely X Ceramic Primer, 3M ESPE AG, Seefeld, Germany), which was then allowed to evaporate for three minutes before being air dried for 30 seconds. The cavity was cleansed, and enamel and dentin were etched with 35% phosphoric acid gel (Primedent) for 15 seconds, rinsed with water for 20 seconds, and then blot-dried with a damp cotton pellet to prepare for cementation. Using a microbrush, SingleBond (3M ESPE) was applied in two coats to both surfaces and allowed to air dry gently for five seconds. The glue was prepared and exposed to a 10-second light cure. Rely X ARC®, a dual-cured resin cement (3M ESPE), was employed for cementation (Figure 6) [5].

**Figure 7:**
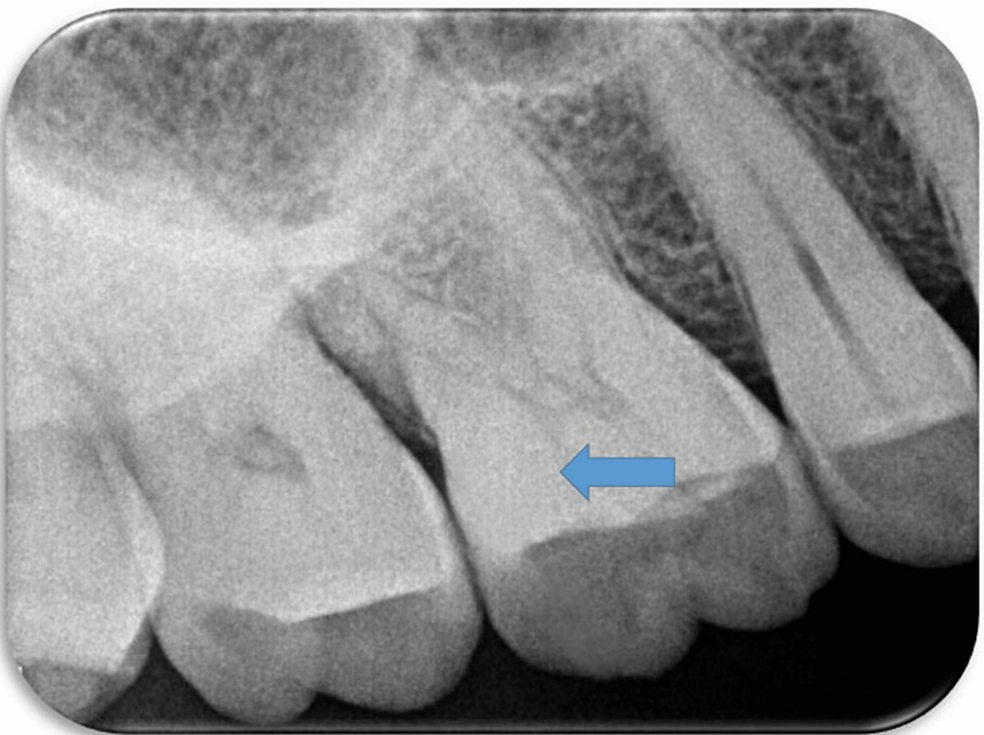
Postoperative radiographic image after onlay cementation

Following cementation, the onlay was examined for occlusion and marginal adaption, and postoperative radiography was performed (Figure 7). At the one-week and one-month follow-ups, the patient did not report any pain, discomfort, or sensitivity when chewing or consuming hot or cold meals.

## Discussion

To ensure the success of ceramic restoration, it is crucial to identify and avoid factors that can impair its clinical performance, such as inferior fabrication, stress tolerance, elastic modulus of the base material, ceramic thickness, cavity preparation, cement selection, adhesion, and surface polishing [5]. Unlike gold-based restorations, ceramic restorations are incredibly delicate prior to bonding and thus require special preparation. Specifically, when preparing a tooth for ceramic restorations, it is essential to avoid regions where internal stress is concentrated, to ensure suitable ceramic thickness and a passive insertion axis [5].

Onlays also offer improved aesthetics, an adhesive bond to enamel and dentine that preserves teeth, reduced plaque accumulation and secondary caries, preservation of soft tissue, ease of prosthodontics, and good access for postoperative care [3]. The projected 10-year survival of ceramic onlays is 92.4%. If the restorations are firmly attached to the tooth, a good clinical success rate for all-ceramic inlays and onlays can be obtained [6-7]. Ceramic inlays and onlays applied with adhesive cement do not fracture and show good clinical performance for up to six months [8]. The survival rate ranged from 91 to 100% according to research conducted over a median period of two to five years. Studies that lasted longer than five years often showed a lower survival rate (71-98.5%). Ceramic onlays have a tendency to perform better clinically than inlays [9]. However, the laboratory charges for inlays and onlays can be as high as those for crowns [10].

## Conclusions

Advances in dental care have led to a decline in dental caries, advancements in dental materials, and increased demand for aesthetically pleasing restorations. In restoring severely damaged posterior teeth, the use of indirect ceramic partial coverage restorations offers a treatment option that can suit patients' aesthetic needs while also restoring the dental form, preserving structurally significant dentine, and protecting the remaining tooth structure.
